# Postnatal outcomes of sonographically suspected isolated congenital lung anomalies

**DOI:** 10.1007/s00383-025-06047-1

**Published:** 2025-06-01

**Authors:** Hanna Heinrich, Ingeborg H. Linskens, Ramon R. Gorter, Matthijs W. N. Oomen, Elisabeth van Leeuwen, Eva Pajkrt

**Affiliations:** 1 Department of Obstetrics and Gynecology, Amsterdam UMC, University of Amsterdam, Meibergdreef 9, 1105 AZ Amsterdam, The Netherlands; 2 Amsterdam Reproduction and Development Research Institute, Amsterdam, The Netherlands; 3 Department of Pediatric Surgery, Amsterdam UMC, University of Amsterdam, Amsterdam, The Netherlands; 4 Amsterdam Gastroenterology Endocrinology Metabolism Research Institute, Amsterdam, The Netherlands

**Keywords:** Congenital lung anomaly, Prenatal diagnosis, Pregnancy outcome

## Abstract

**Introduction:**

The aim was to evaluate the prenatal course, postnatal outcome and diagnostic accuracy of fetuses with a prenatal diagnosis of isolated congenital lung anomalies (iCLA).

**Methods:**

A retrospective cohort is described from the Amsterdam UMC between January 2007 and January 2022. The CPAM volume ratio (CVR) was calculated. The concordance between prenatal diagnosis and lesion progression was compared to postnatal findings. Postnatal surgical interventions were reported.

**Results:**

This study includes 113 prenatal cases of iCLA. Ten percent (10/100 cases with available CVR) progressed into high-risk lesions (CVR > 1.6), with a negative impact on survival. In total, 108 (95.6%) cases resulted in live birth. Acute respiratory distress was observed in 6.5% (7/108). During postnatal follow-up, the lesion was still detectable in 10/15 (66.7%) cases in which complete regression was seen prenatally. Fifty percent (54/108) of the live-born children required surgical management.

**Discussion:**

iCLA has a favorable prognosis in pregnancy, however, close prenatal monitoring is advised. Future parents should be informed about the importance of postnatal follow-up since lesions are often persistent even if prenatal scans may no longer have visualized them, as well as the chance of developing respiratory distress and the probability of undergoing surgery.

## Plain language summary

In cases with a congenital lung anomaly, the CPAM volume ratio (CVR) can be measured prenatally by ultrasound examination as a predictor for a favorable or adverse outcome. A CVR above 1.6 is associated with a high probability of the development of fetal hydrops and adverse outcome. This study demonstrates that the prognosis of an isolated congenital lung anomaly is favorable in most patients. However, it remains challenging to predict acute respiratory distress after birth based on CVR, thus it is suggested that delivery should be within a hospital setting. Subsequently, postpartum imaging should be offered in all patients, as residual lesions often persist, even if prenatal scans may no longer have visualized it.

## Introduction

Congenital lung anomalies (CLA) refer to a heterogeneous group of pulmonary developmental anomalies, including congenital pulmonary airway malformation (CPAM), bronchopulmonary sequestration (BPS), bronchial or larynx atresia and bronchogenic cysts [1, 2]. CPAM is the most common congenital lung anomaly, with the incidence estimated between 1 in 10,000 to 1 in 35,000 live births [3].

Lung development starts from the 4 th week of gestation with the formation of the respiratory diverticulum from the primitive foregut. During pregnancy, due to a process of outgrowth and branching, the primitive foregut differentiates into bronchi, bronchioles and alveoli. From the end of the canalicular phase around 24–26 weeks of gestation, the first gas exchange can take place, allowing the survival of prematurely born fetuses [4, 5]. Congenital lung anomalies may be the result from defective differentiation and budding of the foregut or due to early airway obstruction with secondary dysplastic changes of the lung [1, 3, 6].

Typically, CPAMs are characterized by the vascular origin from the pulmonary circulation, whereas BPS receives blood from the systemic circulation [7, 8]. However, prenatal differentiation between the various types of anomalies can be challenging due to overlapping characteristics or the presence of hybrid CLAs with histologic features of CPAM but with systemic arterial supply characteristic of BPS [1, 2, 9].

Prenatal outcome depends on the growth pattern of the CLA. Large lesions causing compression of the vena cava or high flow from a systemic artery can lead to hydrops fetalis, worsening the prognosis [10]. To prenatally monitor fetuses at risk for developing hydrops, the CPAM volume ratio (CVR) can be measured as a predictor. The CVR quantifies the volume of the lesion relative to the head circumference to correct for gestational age. A CVR above 1.6 is associated with a high probability of development of fetal hydrops and adverse outcome [11–13].

Postnatal management varies based on clinical presentation and final diagnosis after postnatal diagnostic imaging, as CLAs can cause pulmonary hypoplasia and respiratory distress after birth [12]. It has been suggested that in cases of apparent prenatal resolution, approximately 40 percent remains detectable after birth [14]. It is essential to identify persistent lesions, as surgical resection has been suggested even in asymptomatic cases to reduce the risk of recurrent infections and the small risk of malignant transformation [15, 16]. Most children are currently suitable candidates for minimally invasive surgical resection of the lesion [17].

Optimal interpretation of prenatal assessments is important to optimize both prenatal and postnatal care. Therefore, the aim of this study was to evaluate the prenatal and postnatal outcome of prenatally suspected isolated CLA (iCLA). Additionally, to evaluate the concordance between prenatal diagnosis and postnatal findings, the prenatal iCLA progression and specific diagnosis were compared with postnatal findings to evaluate possible discrepancies between prenatal and postnatal assessments.

## Methods

### Design and study population

This is a retrospective cohort study of fetuses with an iCLA at Amsterdam University Medical Centers (Amsterdam UMC, a tertiary referral center for fetal medicine and pediatric surgery) between January 2007 and January 2022. All pregnancies referred to the fetal medicine unit in case of a suspected iCLA were reviewed and cases with a confirmed iCLA after a targeted anomaly scan were included. Cases with suspected multiple congenital anomalies, confirmed aneuploidies or genetic syndromes, maternal age below the age of 18 or unknown pregnancy outcome were excluded. Additionally, cases of suspected larynx atresia were excluded due to distinct ultrasonographic findings compared to other types of iCLA and a more unfavourable prognosis. The study data were collected and managed in an electronic data capture system (Castor EDC). This study has been approved by the medical ethics review committee of the Amsterdam UMC (reference number: W21_361# 21.401).

### Setting

From 2007 onwards, with the introduction of the national screening program, all pregnant women are offered a second-trimester anomaly scan around 20 weeks of gestation in the Netherlands [18, 19]. From September 2021 onwards a first trimester ultrasound scan according to a standardized protocol is offered in a research setting between 12 + 4–14 + 3 weeks of gestation [20]. Ultrasonography in the third trimester of pregnancy is only performed for obstetric indications such as localization of the placenta or assessment of fetal growth and amniotic fluid. If a structural anomaly is suspected during any ultrasound examination, patients are referred to a fetal medicine unit for advanced ultrasound examination, counseling by a multidisciplinary team, invasive diagnostics if requested by parents and delivery if indicated. Amsterdam UMC is a tertiary center for both fetal medicine and pediatric surgery. If a possible iCLA is suspected regular follow-up scans are scheduled, typically every few weeks, to monitor the possible progression of the lesion and fetal condition.

### Data collection

One of the authors (HH) extracted all data from electronic patient files, ultrasound and pediatric databases to extract required data. Prenatal data were obtained, including most likely specific diagnosis, aspect of the cyst on ultrasound based on the prenatal classification of Adzick et al. (microcystic, macrocystic or mixed [21]), maternal age, gestational age at diagnosis and results of prenatal genetic screening if performed. Whenever possible, the CVR was calculated based on the reported measurements obtained during advanced ultrasound examination. This can be obtained by dividing the CPAM volume by the head circumference; CVR = (Length × Height × Width × 0.52 of the lesion)/Head Circumference (cm) [11, 22]. A CVR of less than 1.6 suggests a low risk of developing fetal hydrops in the absence of a large cyst. Fetal hydrops was classified as a collection of interstitial fluid in two or more compartments of the fetal body [23]. Subsequently, a CVR greater than 1.6 is associated with an increased risk of developing fetal hydrops [2, 11]. For all cases, the maximum CVR was reported.

For cases with available data on repeated CVR measurements, prenatal development was assessed using one of the definitions proposed by Peters et al. [24]: Partial regression was characterized by a reduction of the CVR of at least 0.1. Conversely, lesion growth was defined as an increase in CVR from the initial to the last measurement of at least 0.1. Complete regression was defined in cases where an iCLA was no longer detectable on the last ultrasound examination before birth.

In addition, we assessed the prenatal impact of an iCLA on cardiothoracic ratio by comparing the CVR to the cardiothoracic circumference ratio (CT-ratio). The cardiothoracic ratio was calculated by measuring the circumference of the heart relative to the thorax circumference with the electronic ellipse method [25]. Possible complications of the iCLA such as fetal hydrops and polyhydramnios were reported, as were prenatal interventions such as the administration of prenatal corticoids or cyst aspiration.

Perinatal and postnatal data were obtained from electronic patient files, including gestational age at delivery, gender, clinical symptoms (acute respiratory distress within the first 24 h after birth, infections), postnatal imaging and diagnostic imaging techniques (chest X-ray, CT-scan, MRI), postnatal surgical interventions and timing of interventions (in months). Final diagnosis of the iCLA was based on pathology reports in case of surgical resection or based on imaging results in case of expectative management. Respiratory infections were classified as infections within the first year of age with additional diagnostic workup requiring antibiotic treatment as reported in electronic patient files. The minimal follow-up period was one year after birth.

### Statistical analysis

Statistical analysis was performed using the Statistical Package for Social Sciences (SPSS) v. 28. Categorical variables are presented as *n* (%) and continuous variables as mean ± standard deviation (SD) if normally distributed, and as median and interquartile (IQR) if not normally distributed. Independent-samples-*t*-test and Mann–Whitney U test were used for the subgroup analyses of continuous variables, whereas Chi-square analysis or Fisher’s exact test was applied to evaluate categorical variables. *P*-values < 0.05 were considered statistically significant. Comparisons of repeated CVR measurements at different time intervals in pregnancy were done using a Friedman test. Therefore measurements were categorized in three time periods: < 24 weeks, 24–30 weeks, > 30 weeks. When indicated post-hoc pairwise comparisons were conducted using Wilcoxon signed-rank tests with Bonferroni correction to identify specific differences between time points.

Predictive value of the maximum CVR for acute respiratory distress within the first 24 h after birth and surgical intervention was examined using the Receiver Operating Curve (ROC) curve. We considered the overall diagnostic accuracy of the area under the curve (AUC) of 0.5 suggestive of no discrimination, 0.7–0.8 acceptable, 0.8–0.9 excellent and > 0.9 outstanding [26].

## Results

Between January 2007 and January 2022, 122 eligible cases with a prenatally suspected iCLA were referred to the AMC. As pregnancy outcome was unknown in two cases and seven cases did not consent to use of their data for research purposes, a total of 113 cases were included in this study. Demographics and pregnancy outcomes are depicted in Table 1. The median gestational age at detection of the iCLA was 20 + 4 weeks (IQR 20 + 1 − 21 + 2 weeks), with 100/113 (88.5%) of cases identified before 24 weeks of gestation. In the remaining 13 cases diagnosed after 24 weeks of gestation (ranging from 24 + 1–39 + 6 weeks of gestation), no structural anomalies were detected during the second-trimester anomaly scan conducted in line with the Dutch prenatal screening protocol. However, in these cases the iCLA was later identified during growth ultrasounds, each with low CVR. 89/113 (78.8%) of the cases were sonographically classified as a CPAM.

**Table 1:** Patient characteristics

Characteristic	N = 113 Median [IQR], n (%)
Prenatal characteristics	
Maternal age	30.00 [27.0 − 34.0]
Nulliparous	50 (44.2)
GA at diagnosis in weeks	20 + 4 [20 + 1 − 21 + 2]
Fetal male gender	53 (46.9)
Prenatal suspected diagnosis CCAM BPS Bronchogenic cyst	89 (78.8) 21 (18.6) 3 (2.7)
Aspect of lesion	
Microcystic Marocystic Mixed lesion	57 (50.5) 18 (15.9) 38 (33.6)
Maximum CVR	0.51 [0.19–0.94]
Gestational age at maximum CVR	24 + 4 [22 + 3–27 + 4]
Perinatal characteristics	
Livebirth GA at birth in weeks	108 (95.6) 39 + 5 [38 + 4–40 + 5]
Birthweight	3420 [2959–3788]

*IQR* Interquartile range, *n* number, *GA* Gestational age, *CVR* CPAM Volume Ratio

In total, 108/113 (95.6%) cases resulted in live birth, in 4/113 (3.5%) future parents decided to terminate the pregnancy and one pregnancy (0.9%) ended in fetal demise.

### Prenatal monitoring and outcome

Data on at least one CVR measurement was available for 100 cases. Median maximum CVR was 0.51 (median 0.19–0.94) at a median of 24 + 4 weeks (IQR 22 + 3–27 + 4 weeks) of gestation. Ten (10%) cases progressed into a high-risk lesion with a CVR exceeding 1.6. Lesions with a CVR > 1.6 were significantly associated with the development of mediastinal shift of the heart, polyhydramnios and fetal hydrops, which negatively impacted the chance of survival (Table 2). Prenatal intervention was pursued in 9/10 of the cases with CVR > 1.6. In one case, a macrocystic cyst was drained at 17 weeks of gestation. In the remaining eight cases, systemic corticosteroids were administered to the mother at a mean gestational age of 23 + 4 weeks (SD 15 days). Six of the ten (60%) cases resulted in live birth, whereas three pregnancies were terminated and one ended in fetal demise.

**Table 2:** CVR and outcome

	CVR ≤ 1.6 (*n* = 90)	CVR > 1.6 (*n* = 10)	*P* value
Mediastinal shift	47 (52.2)	10 (100.0)	0.004*
Fetal hydrops fetalis	0 (0)	2 (20.0)	0.009*
Polyhydramnios	3 (3.3)	5 (50.0)	< 0.001*
Fetal intervention	1 (1.1)	9 (90.0)	< 0.001*
Neonatal respiratory distress	4 (4.4)	3 (30.0)	0.02*
Postnatal surgical intervention	46 (51.1)	6 (60.0)	0.742
Live birth	89 (98.9)	6 (60.0)	< 0.001*

*CVR* Congenital cystic adenomatoid malformation volume ratio

*P-values < 0.05 were considered statistically significant

Of the iCLA with a CVR < 1.6 (*n* = 90), live birth occurred in 89/90 (98.9%) cases, which was significantly higher compared to iCLA with a CVR > 1.6 (*p* < *0.001). O*ne case (2.1%) with CCAM and a CVR of 1.41 was terminated. Another case with a CVR of 0.41 developed ascites, for which prenatal corticosteroids were administered. Subsequently, ascites resolved after the corticosteroid treatment and the pregnancy resulted in a live birth.

The CT-ratio of the cases with available measurements (*n* = 69) at the time of the maximum CVR measurement in lesions with a CVR > 1.6 was significantly lower compared to cases with a CVR < 1.6 (median CT-ratio 0.41 vs. 0.47).

**Fig. 1:**
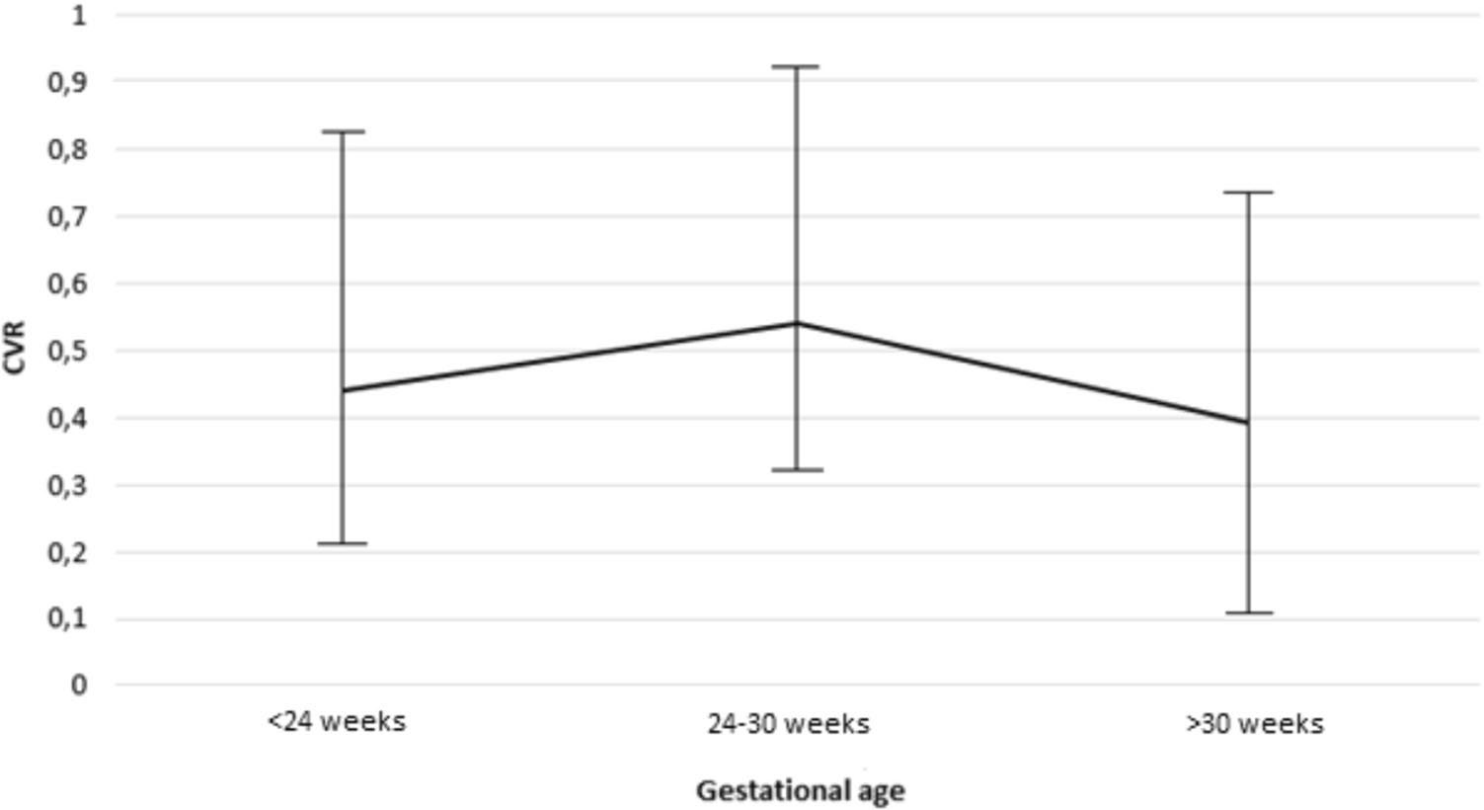
Repeated CVR measurements

When evaluating the CVR measurements across multiple time points in ongoing pregnancies with available data (*n* = 46), the highest median CVR was observed between 24–30 weeks of gestation (median CVR 0.54, IQR 0.32–0.91) (Fig. 1). Friedman’s test revealed a significant overall difference across the multiple measurements (*p* < 0.05). Subsequent Wilcoxon-rank test revealed a significant difference between the median CVR between 24 and 30 weeks of gestation and those after 30 weeks of gestation (median CVR 0.39, IQR 0.11–0.77) (*p* < *0.001)*. However, there were no statistically significant differences in median CVR before 24 weeks of gestation (median CVR 0.44, IQR 0.21–0.82) compared to subsequent measurements in advancing pregnancy (< 24 weeks vs. 24–30 weeks *p 0.061,* < 24 weeks vs. > 30 weeks *p 0.334).*

Throughout the prenatal follow-up complete regression of the lesion was suspected in 21 (18.6%) cases, as the iCLA could no longer be visualised. Growth of the iCLA was observed significantly more often in macrocystic lesions compared to microcystic lesions (50% vs. 18%, *p 0.031*), but was not significantly higher compared to mixed lesions (50% vs. 28.6%, *p 0.195*) (Table 3). A higher trend in lesion reduction or complete regression was observed in microcystic lesions, but this was not statistically significantly different compared to the other types of lesions.

**Table 3:** Prenatal development of iCLA based on CVR

	Total *n* = 92	Microcystic *n* = 50	Macrocystic *n* = 14	Mixed lesion *n* = 28
Increase	24 (26.1)	9 (18.0)	7 (50.0)	8 (28.6)
Stable	27 (29.3)	15 (30.0)	2 (14.3)	10 (35.7)
Decrease	20 (21.7)	11 (22.0)	2 (14.3)	7 (25.0)
Complete regression	21 (22.9)	15 (30.0)	3 (21.4)	3 (10.7)

Of the 108 liveborn cases, median gestational age at birth was 39 + 5 (IQR 38 + 4–40 + 5) weeks. Six neonates were born prematurely, ranging from 28 to 36 weeks. Normal birth weight percentiles were observed in 103/108 (95.4%). Neonates presented with acute respiratory distress after birth in 7/108 (6.5%). Acute respiratory distress was observed in 3/6 (50%) of live-born cases with a maximum CVR > 1.6. Of the four cases with a CVR < 1.6, one case with suspected complete regression of the iCLA suffered from respiratory distress after umbilical compression due to a tight loop of umbilical cord around the neck and one case was born prematurely. The latter two cases that suffered from respiratory distress requiring respiratory support had a maximum CVR of 0.38 and 0.47, of which one was delivered via caesarean section. During postnatal follow-up, respiratory infections requiring antibiotic treatment within the first year were reported in 12% (13/108). One premature born neonate (29 weeks of gestation) died within the first week of live due to the complications of prematurity. Overall survival of the entire cohort was 94.7%.

### Concordance between prenatal and postnatal diagnosis

**Fig. 2:**
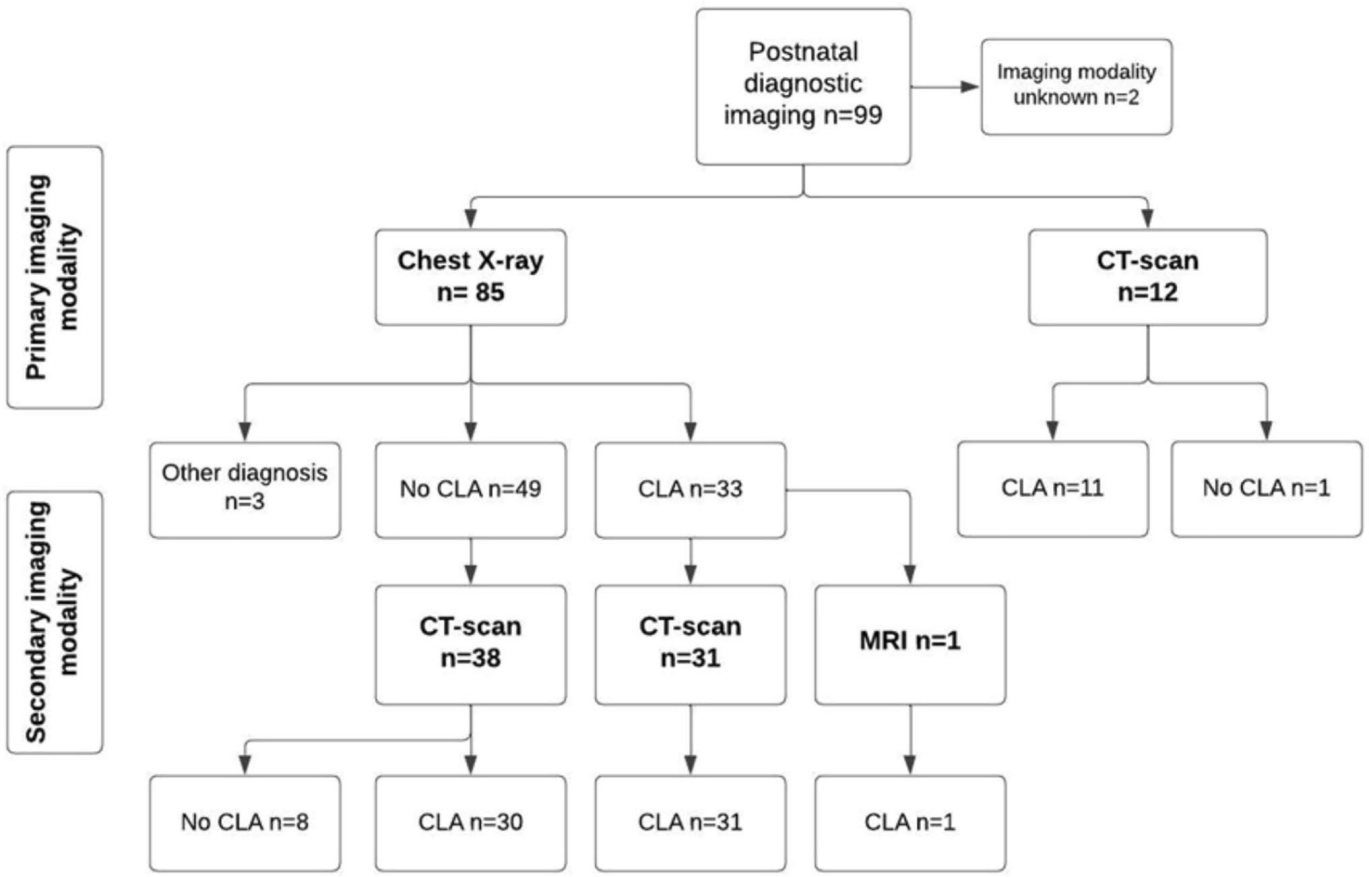
Postnatal diagnostic imaging

Of the live-born cases 99/108 had available data on postnatal imaging (Fig. 2). In two cases, the postpartum diagnosis was obtained at another hospital facility, but the diagnostic imaging modality used to obtain the diagnosis was not reported back. In 75/99 (75.8%) cases a persistent iCLA was found. The lung lesion could no longer be detected in 20 (21.2%) cases. In four cases, all with normal lung development on postnatal imaging, a different anomaly was found than suspected prenatally. An abdominal cyst was present in two cases, a diaphragmatic hernia was detected in one case and one case had a diaphragmatic paralysis. In addition, in one case a diaphragmatic hernia and a BPS were diagnosed in the same neonate. Of the cases with postnatal imaging, 85/99 initially received a chest X-ray, of which a lung lesion was suspected in 33/85 (38.8%). However, of the 49 cases where X-ray did not suggest an iCLA, subsequent CT-scan performed in 38 cases revealed a persistent iCLA in 30/38 cases (78.9%). In cases with prenatal suspected decrease of the iCLA during pregnancy, the lesion was still visible in cases with postnatal follow-up in 30/35 (85.7%). Among the cases with available follow-up where complete prenatal regression was suspected, 10 out of 15 (66.7%) cases were diagnosed with a persistent iCLA after birth.

The agreement between the prenatal and postnatal specific diagnosis of the iCLA was confirmed in 37/75 (49.3%) of the persistent lung anomalies (Appendix 1). In 11/75 (14.7%) a bronchial atresia was diagnosed, which was not considered the most likely diagnosis prenatally in any of these cases. Of these cases with bronchial atresia 10/11 cases were microcystic lesions. None of the lesions were classified as a malignant lesion postpartum.

### Postnatal surgery

Of the live-born children, 54/108 (50%) underwent surgical treatment at a median age of 10 months (IQR 6–14 months). The impact of COVID-19, led to extended waiting periods before surgical treatment in some cases. All of the six live-born cases with a prenatal CVR exceeding 1.6 underwent surgery treatment between 1 and 6 months after birth. In patients that underwent surgical treatment, the median CVR was found to be significantly higher compared to the cases with expectative management (median CVR 0.66 vs 0.25, *p* < *0.001*). Surgeries were predominantly performed in cases with CPAM (28/32, 87.5%), BPS (17/17, 100%) and hybrid CLAs (6/7, 85.7%). Only one of the eleven cases (9.1%) with bronchial atresia required surgery due to air trapping.

ROC-curve analysis demonstrated an AUC of 0.72 (95% confidence interval [CI]: 0.62–0.83) for the predictive value of CVR within our cohort for surgical treatment, with an optimal cut-off CVR value of 0.53 (sensitivity 61.5%, specificity 71.8%). Furthermore, the ROC curve analysis indicated the CVR could not predict the chance of respiratory distress in the first 24 h after birth (AUC 0.61).

## Discussion

Within our cohort, we evaluated the outcome of fetuses prenatally suspected of an iCLA. Our findings demonstrate that overall survival rates in cases of iCLA, especially with a low CVR, is high (94.7%). Our study confirms that a CVR > 1.6 negatively impacts fetal outcomes, as live birth in this group was 60 percent compared to 98.9 percent in the group with a CVR < 1.6. Given that the majority of these anomalies are detected before 24 weeks of gestation, there is a crucial role for optimal recognition of the prenatal features of these anomalies to optimize further counseling. In more than 65 percent of cases a residual lung lesion was diagnosed postpartum, even though complete regression of the lesion was suspected prenatally.

During postpartum follow-up of the suspected pulmonary anomalies, a persistent CLA was identified in 75/99 (75.8%) of the cases. Even in cases where reduction in size or even complete regression was suspected, persistent CLAs were observed on postpartum imaging in 85.7%. In addition, in 30/38 (78.9%) cases where chest X-ray did not identify a CLA, subsequent CT-scan did reveal a persistent anomaly. This is emphasized by current literature, which suggests CT- imaging as the preferred imaging modality for diagnosis of congenital lung anomalies [27, 28]. In asymptomatic cases, this is scheduled around three to four months after birth. Hence, postpartum diagnostic imaging with CT-scan should be recommended for all fetuses with suspected iCLA to verify complete resolution of the anomaly, as asymptomatic lesions could still become infectious. In addition, a low risk for malignant conversion of the cystic wall has been described, predominantly in cases of CPAM associated with *KRAS* mutations. [6, 16, 29–31]. Bronchial atresia was never considered as the most likely diagnosis prenatally, yet postpartum imaging revealed 11 (14.7%) cases. As the majority of cases with bronchial atresia in our cohort did not require surgical treatment, enhanced prenatal diagnosis could potentially improve counseling in the prognosis of surgical treatment. Available case reports on fetal bronchial atresia described an echogenic mass with a microcystic pattern [32, 33]. In our cohort, 10/11 cases with bronchial atresia exhibited a lesion with a microcystic aspect, potentially leading to the misinterpretation of the hyperinflated obstructed segment of the lung for a CPAM or BPS. Additionally, the lower prevalence of bronchial atresia compared to CPAM and BPS may reduce the probability of prenatally identifying bronchial atresia as the most likely diagnosis [8, 34]. Moreover, as described in previous literature, the heterogeneous spectrum of cystic lung anomalies may result in the reclassification of prenatally suspected CPAM to BPS or a hybrid lesion [2, 35]. Parents should be aware of this reclassification, but as the majority of the surgical interventions within our cohort were performed in cases of CPAM, BPS or hybrid lesions (51/54, 94.4%), this is likely to have a small impact on the expected postnatal interventions.

When assessing the cases with available multiple measurements, a trend was seen in the maximum median CVR between 24 and 30 weeks of gestation. This is consistent with prior research, which has described a plateau stage at the end of the second trimester is, after which the lesion either stops growing or decreases in size relative to the growth of the fetus [24, 36–38]. This may also explain the delayed diagnosis after 24 weeks of gestation in 13/113 cases within this cohort, as lesion growth can increase the detectability of the CLM. A significant decrease was seen in the CVR measurements taken after 30 weeks of gestation, showing the improvement of the lesion size before birth. However, reduced acoustic windows, fetal position and decreased contrast between the lesion and surrounding lung tissue due to increased echogenicity of the normal lung tissue in advanced pregnancy may hinder optimal evaluation of the lesion in the third trimester [39].

As described in previous literature, we also observed a predictive value of a high CVR on prenatal complications in our cohort, which negatively impact fetal outcome [11, 40, 41]. In addition, we measured a significantly smaller CT-ratio in cases with a higher CVR. However, we were unable to demonstrate the predictive value of CVR for postpartum outcome within our cohort. The CVR and corresponding CT-ratio in our cohort did not demonstrate a clear influence on the risk of acute respiratory distress, as only half (3/6) of the live-born cases with CVR above 1.6 presented with acute respiratory distress. A possible explanation is the maximum CVR before the third trimester, which was in correspondence with previous literature, which may allow for compensatory lung development after regression of the lesion [24, 42]. An additional four cases with a CVR below 1.6 presented with acute respiratory distress. This emphasizes the recommendation for a hospitalized delivery, even in cases where regression of the anomaly is suspected, as cases with a low CVR can still experience respiratory distress. This is of importance in the Dutch obstetric health care system as the Netherlands has the highest rate of home deliveries in Europe [43]. However, other factors such as prematurity or delivery via cesarean could have increased the risk of the need for respiratory support as well in the described cases. This complicates the assessment of whether acute respiratory distress can be solely attributed to the CLA. Although we did observe a significantly higher CVR in children who underwent surgical intervention, determining an optimal cut-off value for surgical intervention proved challenging due to a suboptimal Area Under the Curve (AUC) in our Receiver Operating Characteristic (ROC) analysis (AUC 0.72). The lack of predictive value of the CVR within this cohort for surgical intervention may be a result of limited sample size, in specific of the high-risk lesions, as only six live-born cases in our cohort had a prenatal CVR > 1.6. Among the 54 surgical cases within the cohort, all cases with a prenatal CVR exceeding 1.6 underwent surgical correction. However, the majority of cases that underwent surgical correction had a CVR < 1.6 (48/54), possibly diminishing the predictive value of CVR for surgical intervention.

As data on this study were collected from 2007 onwards, the detection of congenital lung anomalies subsequent to the introduction of the second trimester anomaly scan in the Dutch screening program is well represented within our study. However, as bigger anomalies will be detected more easily during the anomaly scan, this could result in some selection bias, as the prevalence of low-risk lesions in the whole population might even be higher than is represented within this study. Consequently, the prognosis for isolated congenital lung anomalies may even be more favorable than was demonstrated within this study. The primary limitation of this study is its retrospective nature. Absence of a standardized protocol for congenital lung anomalies led to variations in gestational age during prenatal follow-up ultrasound examinations and the frequency of visits. In addition, missing data on CVR due to the lack of the three required measurements (transverse, sagittal and anteroposterior diameter) of the lesion in two orthogonal planes, as well as the limited availability of multiple measurements, possibly limits more precise predictions concerning the predictive value of CVR for postnatal outcomes. Therefore, if a congenital lung abnormality is suspected, measurement of the CVR at every prenatal follow-up should be emphasized. This approach facilitates monitoring of the lesions and the progression or regression in relation to the fetal size.

## Conclusion

This study shows that children with an isolated congenital lung anomaly, particularly those with a low CVR, have a very good prognosis. A delivery in a hospital is indicated in cases with a suspected lung anomaly, even if the lesion appears to be small or to have regressed completely, as it remains challenging to predict the chance respiratory distress based on CVR. Subsequently, postpartum imaging by CT-scan should be offered in all cases, as residual lesions often persist and X-radiology may not identify the lesion.

## Data Availability

All data generated or analyzed during this study are included in this article. Further enquiries can be directed to the corresponding author.

## Appendix 1: Postnatal diagnosis

**Table:**

*N* = 99	Prenatal diagnosis		
Postnatal follow-up	CPAM (*n* = 76)	BPS (*n* = 20)	Bronchogenic cyst (*n* = 3)
No lesion	13 (17.1)	6 (30.0)	1 (33.3)
Persistent lung lesion	56 (73.7)	12 (60.0)	1 (33.3)
CPAM	30	2	–
BPS	9*	8	–
Hybrid lesion	7	–	–
Bronchial atresia	10	1	–
Bronchogenic/pulmonary cyst	–	1	1
Other lung anomaly	5 (6.6) Congenital lobar Emphysema *n* = 4 Atelectasis *n* = 1	1 (5.0) Bronchiectasis *n* = 1	–
Other anomaly	2 (2.6) Diaphragm. hernia *n* = 2* Diaphragm. paralysis *n* = 1	1 (5.0) Abdominal cyst *n* = 1	1 (33.3) Abdominal cyst *n* = 1

*One case of diaphragmatic hernia and BPS in the same neonate

*CPAM* Congenital pulmonary airway malformation, *BPS* Bronchopulmonary sequestration
